# Elevated Expression of *AXL* May Contribute to the Epithelial-to-Mesenchymal Transition in Inflammatory Bowel Disease Patients

**DOI:** 10.1155/2018/3241406

**Published:** 2018-07-22

**Authors:** Éva Boros, Zoltán Kellermayer, Péter Balogh, Gerda Strifler, Andrea Vörös, Patrícia Sarlós, Áron Vincze, Csaba Varga, István Nagy

**Affiliations:** ^1^Institute of Biochemistry, Biological Research Centre, Hungarian Academy of Sciences, Szeged, Hungary; ^2^Department of Immunology and Biotechnology, University of Pécs, Pécs, Hungary; ^3^Lymphoid Organogenesis Research Group, Szentágothai János Research Center, University of Pécs, Pécs, Hungary; ^4^Seqomics Biotechnology Ltd., Mórahalom, Hungary; ^5^ATGandCo Biotechnology Ltd., Mórahalom, Hungary; ^6^1st Department of Internal Medicine, University of Pécs, Pécs, Hungary; ^7^Department of Physiology, Anatomy and Neuroscience, University of Szeged, Szeged, Hungary

## Abstract

Understanding the molecular mechanisms inducing and regulating epithelial-to-mesenchymal transition (EMT) upon chronic intestinal inflammation is critical for understanding the exact pathomechanism of inflammatory bowel disease (IBD). The aim of this study was to determine the expression profile of TAM family receptors in an inflamed colon. For this, we used a rat model of experimental colitis and also collected samples from colons of IBD patients. Samples were taken from both inflamed and uninflamed regions of the same colon; the total RNA was isolated, and the mRNA and microRNA expressions were monitored. We have determined that AXL is highly induced in active-inflamed colon, which is accompanied with reduced expression of AXL-regulating microRNAs. In addition, the expression of genes responsible for inducing or maintaining mesenchymal phenotype, such as SNAI1, ZEB2, VIM, MMP9, and HIF1*α,* were all significantly induced in the active-inflamed colon of IBD patients while the epithelial marker E-cadherin (CDH1) was downregulated. We also show that, *in vitro*, monocytic and colonic epithelial cells increase the expression of *AXL* in response to LPS or TNF*α* stimuli, respectively. In summary, we identified several interacting genes and microRNAs with mutually exclusive expression pattern in active-inflamed colon of IBD patients. Our results shed light onto a possible *AXL*- and microRNA-mediated regulation influencing epithelial-to-mesenchymal transition in IBD.

## 1. Introduction

Inflammatory bowel disease (IBD) is a group of multifactorial disorders characterized by chronic inflammation along the digestive tract, and in combination with lifestyle, it is associated with genetic and environmental factors [1, 2]. IBD comprises two main types of intestinal inflammation: Crohn's disease (CD) and ulcerative colitis (UC). While CD may occur from the mouth to the anus, UC is limited to the colon [3]. Of note, an increasing incidence of IBD has been reported worldwide, especially in economically well-developed countries [4]. As a life-long disease, the main symptoms of IBD—such as abdominal pain, diarrhea, and fatigue—are highly reducing the quality of life; in addition, prolonged inflammation increases the risk of colitis-associated colorectal cancer (CRC) [5]. Inflammatory conditions, among others, play roles at different steps of tumor development such as initiation, invasion, metastasis, and epithelial-to-mesenchymal transition (EMT) [6]. The aberrantly functioning common signaling pathways and molecules are able to link immune response and cancer progression to each other [7]. In line with this, defects in immune tolerance in the gut against commensal microorganisms or insufficient negative immune regulation may cause robust inflammation leading to colitis-associated colorectal cancer [8].

TAM receptors are known, among others, as pleiotropic negative regulators of the immune system [9, 10]. This family of cell surface transmembrane receptors has three members: TYRO3, AXL, and MERTK. Their transmembrane domain is connected to the extracellular domain comprising tandem repeats and to the cytoplasmic protein tyrosine kinase domain, which is responsible for signal transduction after ligand-activated receptor dimerization [11]. The two ligands that bind to and activate TAM receptors are growth arrest-specific gene 6 (GAS6) and protein S (PROS) which have distinct expression patterns in mammalian tissues [12]. Apart of inhibiting inflammation TAM receptor signaling has been shown to have important regulatory roles in vascular smooth-muscle homeostasis, erythropoiesis, and cancer development [12]. In addition, TAM receptors are promoting inflammatory responses; as phagocytic receptors, they also have a role in the clearance of apoptotic cells and in stimulating the maturation of natural killer cells [9, 13]. Importantly, AXL impinges on cell motility and invasion compared to TYRO3 and MERTK [14, 15], while TYRO3 acts as an inhibitor of type 2 immunity during allergic reactions [16]. TAM receptors AXL and MERTK are also known as protooncogenes: they are highly expressed in numerous tumor cells [17]. Hence, the therapeutic targeting of AXL and MERTK kinases is considered an anticancer strategy: small-molecule inhibitors as well as biologics are in preclinical development [18].

The increased expression of AXL has been reported in several cancer types, such as lung, breast, and colorectal as well as head and neck cancer [11]. Furthermore, the AXL expression level inversely correlates with survival; hence, it is considered as a strong predictor of poor clinical outcome [19]. AXL has been shown to act through several signaling pathways: activation of NF-*κ*B and JAK/STAT leads to the regulation of immune response and suppression of inflammation [12]; in contrast, the activation of transcriptional factors SNAI1/2, ZEB2, or TWIST stimulates EMT [20, 21]; moreover, AXL mediates tumor invasion by the regulation of matrix metalloproteinase-9 (MMP9) expression [22]. During EMT, transcription factors induce the breakage of cell–cell connections through the suppression of epithelial marker E-cadherin (CDH1), while expression of mesenchymal markers vimentin (VIM) and MMP9 induces reorganization of the extracellular matrix giving rise to mesenchymal phenotype [23]. Inflamed tissues and tumor microenvironment are characterized by limited supplies of oxygen, which provokes the expression of hypoxia-inducible factor 1 alpha (HIF1*α*) [24]. As a consequence, HIF1*α* induces a NOTCH signaling pathway, critical in the regulation of EMT-related transcriptional factors, and also enhances the expression of AXL [14, 25].

Dysregulated expression of miRNAs has been described in various cancer types where miRNAs can act as tumor suppressors or oncogenes [26]. Several miRNAs, such as miR-34a, miR-199a, and miR-92b, are known to posttranscriptionally regulate the expression of *AXL* [14, 27–29]. For example, miR-34a regulates AXL expression in lung as well as in head and neck cancer cells, where high tumor *AXL* mRNA expression associates with poor survival [27, 30]; miR-34a and miR-199a reduce the expression of AXL in non-small-cell lung cancer and colorectal cancer cell lines [28]; and miR-92b reduces the amount of *AXL* in fibroblasts [29, 31].

Defining molecular relationships between chronic inflammation and EMT is critical for understanding the progression of IBD and colitis-associated CRC. We have previously shown that, among others, EMT is activated due to the opposed expression profile of genes and their regulating microRNAs at the site of inflammation in a rat model of experimental colitis [32]. Here, we show that TAM family receptor Axl is highly induced in experimental colitis, which is accompanied with reduced expression of Axl-regulating microRNAs. In addition, we have now tested the expression of the selected genes and microRNAs on samples derived from colons of IBD patients and have determined that the expression pattern of all tested molecules is the same as in the case of a rat model of experimental colitis.

## 2. Materials and Methods

### 2.1. In Vivo Rat Model and Sample Collection

Experimental colitis in rats was induced by 2,4,6-trinitrobenzene sulphonic acid (TNBS) as described previously [32]. Briefly, male Wistar rats were randomly divided into two groups: the first group served as control (vehicle-treated hence noncolitis-induced) and the second group was induced by TNBS (colitis-induced) based on the method described by Morris et al. [33]. 72 hours after the treatment, all animals were sacrificed, and distal colons were removed. In the case of the control group, samples were taken from random colon sections; samples from colitis-induced animals were taken from inflamed colon region as well as from nonadjacent uninflamed region. All samples were kept in a TRIzol reagent (Thermo Fisher) at −80°C.

### 2.2. Patients

Colonic biopsies were obtained from 10 consenting patients with IBD (6 females and 4 males; median age 39 years, 28–48 years) undergoing colonoscopy for diagnostic purposes approved by the Hungarian Medical Research Council's Committee of Scientific and Research Ethics (ETT TUKEB). Sample collection and classification were performed according to the disease status of patients, active/relapsing or inactive/remission phase. Furthermore, samples from relapsing patients were subdivided as uninflamed or inflamed according to the status of the colon tissue.

### 2.3. Cell Culture Conditions and Stimulations

The human THP-1 monocytic cells and HT-29 colonic epithelial cells were maintained in a RPMI-1640 (Gibco) or DMEM (Gibco) medium, respectively, supplemented with 10% fetal bovine serum (FBS; Gibco) and 1% antibiotic/antimycotic solution containing 10,000 units/ml of penicillin, 10,000 *μ*g/ml of streptomycin, and 25 μg/ml of amphotericin B (Gibco). Cells were cultured at 37°C in an atmosphere of 5% (*v*/*v*) CO_2_ in air. In order to activate THP-1 cells, the culture medium was additionally supplemented with 5 ng/ml phorbol myristate acetate (PMA; Sigma) for 48 h prior to the experiment. For TNF*α* or LPS treatment, cells were seeded on six-well cell culture plates (Sarstedt) and stimulated with 1 *μ*g/ml LPS (Sigma) or 10 ng/ml TNF*α* (R&D Systems) for the indicated time, followed by RNA extraction.

### 2.4. Extraction of Total RNA, Reverse Transcription, and Quantitative Real-Time PCR (QRT-PCR)

Samples from rat colons were homogenized in a TRIzol reagent by an ULTRA-TURRAX T-18 (IKA) instrument as described previously [32]. 0.1 ml of chloroform (Sigma-Aldrich) was added to a 0.3 ml homogenized sample with vigorous vortexing. The samples were centrifuged at 13000 rpm for 10 minutes. The total RNA was then extracted from the upper aqueous phase. RNeasy Plus Mini Kit (Qiagen) was used to purify the total RNA from rat colon samples, as well as human THP-1 and HT-29 cells, according to the manufacturer's protocol. Human colonic biopsies were obtained by experienced gastroenterologists at the 1st Department of Internal Medicine, University of Pécs, in accordance with the guidelines set out by the Medical Research Council of Hungary. The total RNA from human biopsies was isolated using NucleoSpin RNA Kit (Macherey-Nagel) according to the manufacturer's protocol. The quality and the quantity of the extracted RNAs were determined by TapeStation (Agilent) and Qubit Fluorometer (Thermo Fisher).

Reverse transcription and QRT-PCR were performed as described previously [32]. All measurements were performed in duplicate with at least two biological replicates. Specific exon spanning primer sets and TaqMan Gene Expression Assays (Thermo Fisher) are listed in Tables 1 and 2, respectively. The ratio of each mRNA relative to the 18S rRNA was calculated using the 2^−ΔΔ*C*T^ method. The specific miRNA assays (Thermo Fisher) are shown in Table 3. The ratio of each miRNA relative to the endogenous U6 or RNU48 snRNA for rat or human, respectively, was calculated using the 2^−ΔΔCT^ method.

### 2.5. Tissue Sections and Immunofluorescent Labelling

Rat colons were embedded in Technovit 7100, and tissue sections of 7 *μ*m thickness were cut with Reichert Jung 1140 Autocut microtome for immunofluorescent staining. Tissue sections were then blocked for 20 minutes in PBS (Gibco) containing 5% fetal bovine serum (FBS; Gibco) and 0.1% TritonX (Sigma). Next, sections were incubated overnight with mouse anti-Axl (Santa Cruz) primary antibody (1 : 100 dilution) or isotype-matched negative control antibody, washed three times with PBT (PBS containing TritonX) for 10 min each, incubated with FITC conjugated anti-mouse IgG (Sigma) secondary antibody (1 : 250 dilution) for 90 minutes, and washed three times with PBT (10 minutes each). Finally, samples were washed with PBS containing 4′,6-diamidino-2-phenylindole (DAPI; 1 : 10000 dilution) and mounted in Citifluor mounting media (Citifluor Ltd.). Samples were analyzed using epifluorescent illumination of the Axiovision Z1 fluorescent microscope (Zeiss), and images recorded by Axiovision software.

### 2.6. Statistical Analysis and Data Representation

Statistical evaluations were performed using the IBM SPSS Statistics program for Windows. Graphs were plotted with GraphPad Prism 6 software. Quantitative data are presented as the mean ± SEM, and the significance of difference between sets of data was determined by one-way analysis of variance (ANOVA) following LSD post hoc test; a *p* value of less than 0.05 was considered significant.

## 3. Results and Discussion

### 3.1. Expression Pattern of TAM Receptors and Their Ligands in Rat Experimental Colitis

**Figure 1:**
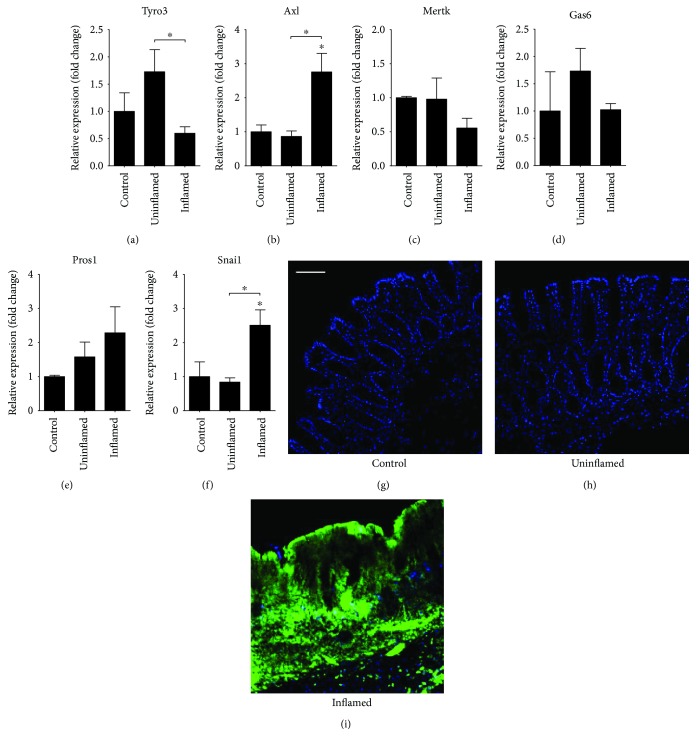
Diverse expression patterns of TAM receptors and their ligands at the site of colon inflammation in rat experimental colitis. The relative gene expression of TAM receptors *Tyro3* (a), *Axl* (b), and *Mertk* (c) and their ligands *Gas6* (d) and *Pros1* (e) as well as the EMT-inducer *Snai1* (f) is shown from control (left columns, *n* = 2), uninflamed (middle columns, *n* = 6), and inflamed (right columns, *n* = 6) rat colon sections. (g–i) Protein expression of Axl on rat colon sections. Data on (a–f) are presented as the mean ± SEM; ^∗^*p* < 0.05. Scale bar for (g–i): 100 *μ*m; blue: DAPI; green: Axl protein.

Increasing evidence suggests the malfunction in the negative regulation of immune response, thereby robust and prolonged inflammation leads to colitis-associated CRC [11, 12]. Since TAM receptors are involved in both negative regulation of the immune response and in tumorigenesis, we first sought to analyze their expression level in rat experimental colitis. We have determined that the gene expression of *Axl* is significantly induced in inflamed regions of the colon as compared to both controls and uninflamed regions (Figure 1(b)). In contrast, we could not detect any significant change in gene expression of neither receptors *Tyro3* and *Mertk* (Figures 1(a) and 1(c)) nor ligands *Gas6* and *Pros1* (Figures 1(d) and 1(e)) as compared to controls. Next, we performed immunofluorescent labelling using anti-Axl mAb on tissue sections. In line with the increased gene expression of *Axl*, we detected a marked increase of Axl protein in the lamina propria of inflamed regions (Figures 1(g)–1(i)). Given that AXL exerts a wide array of functions including immune regulation, clearance of dead cells, cell survival, proliferation, migration, and adhesion [34], it is difficult to conclude which process is affected by its increased expression in experimental colitis.

### 3.2. Association of Axl with EMT in Rat Experimental Colitis

AXL is associated with mesenchymal phenotype [35] with increased expression in non-small-cell lung cancer cell lines [36]. The knockdown of *AXL* in MDA-MB-231 breast cancer cell line reduced the expression of mesenchymal marker vimentin [37]. In contrast, *AXL* overexpression led to the elevated expression of EMT inducers, such as SNAI1, and repression of CDH1 in breast cancer stem cells [21]. We have previously reported the induction of EMT-related genes including *Vim* and *Zeb2* in the inflamed rat colon samples accompanied with decreased expression of epithelial marker E-cadherin (*Cdh1*) [32]. Here, we show that in parallel with increased *Axl* expression (Figure 1(b)), *Snai1* is also upregulated in inflamed regions of rat colons (Figure 1(f)). These data suggest that the increased expression of *Axl* in rat experimental colitis may trigger the expression of EMT inducers *Zeb2* and *Snai1*, thereby linking increased *Axl* expression to EMT.

### 3.3. AXL and EMT Genes Show Altered Expression Pattern in IBD Patients

In relapsing disorders, such as IBD, active and inactive/remission intervals alternate. In the active phase, symptoms manifest and inflammation flames up with damaged and phenotypically intact regions sporadically following each other along the gut lumen [38]. We have shown that in the case of rat experimental colitis, several genes and miRNAs show distinct expression patterns between uninflamed and inflamed regions of colitis-induced colons [32]. Thus, we have collected colonic biopsies from IBD patients similarly to our sampling model applied in the case of rat experimental colitis. Two samples from the colons of IBD patients in active phase were collected: one sample corresponds to the phenotypically uninflamed region while the other to the severely inflamed region, referred to as active-uninflamed and active-inflamed, respectively.

**Figure 2:**
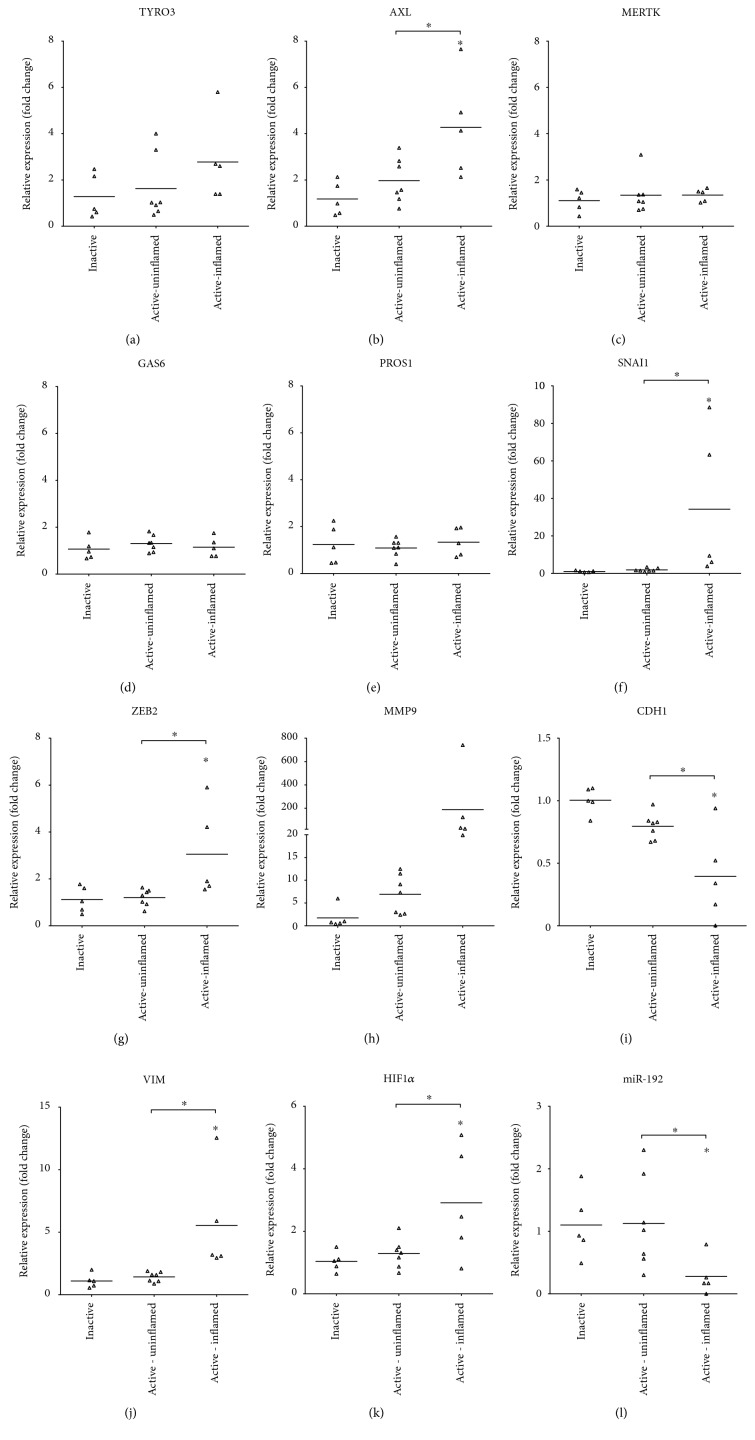
Distinct expression of genes and microRNA regulating epithelial-to-mesenchymal transition in IBD patients. The relative gene expression of TAM receptors *TYRO3* (a), *AXL* (b), and *MERTK* (c); their receptors *GAS6* (d) and *PROS1* (e); and genes involved in epithelial-to-mesenchymal transition *SNAI1* (f), *ZEB2* (g), *MMP9* (h), *CDH1* (i), *VIM* (j), and *HIF1α* (k) as well as microRNA miR-192 (l) are shown from inactive (left, *n* = 5), active-uninflamed (middle, *n* = 7), and active-inflamed (right, *n* = 5) colon samples of IBD patients. Dots and lines represent individual values and the mean, respectively; ^∗^*p* < 0.05.

Next, we determined the expression of TAM receptors and their ligands *GAS6* and *PROS1* as well as genes involved in EMT on these samples. In the case of TAM receptors and their ligands, only *AXL* was significantly induced in the active-inflamed colon biopsies compared to both the inactive and the active-uninflamed samples (Figures 2(a)–2(e)). Recent reports have shown that AXL regulates EMT through the activation of SNAI1, ZEB2, and MMP9 in several human malignancies [20, 21]. In line with these data, all three genes were significantly induced in the active-inflamed regions of relapsing IBD patients (Figures 2(f)–2(h)). These data are in agreement with those observed in rat experimental colitis (Figure 1(f) [32]). The main role of the increased ZEB2 in EMT is the suppression of E-cadherin coding *CDH1* gene [39]. In line with this and our earlier data [32], the expression of *CDH1* was significantly downregulated in active-inflamed regions (Figure 2(i)). Elevated MMP9 and vimentin are responsible for the formation of mesenchymal phenotype and the maintenance of microenvironment by, among others, inducing HIF1*α* [23, 24]. We have determined that all these genes were upregulated in the active-inflamed regions of relapsing IBD patients (Figures 2(h), 2(j), and 2(k)) corroborating earlier data from rat experimental colitis [32]. Taken together, these data suggest that EMT takes place in the active-inflamed regions of IBD patients in which *AXL* may play a regulatory role.

### 3.4. Dysregulated MicroRNA Expression Drives EMT in IBD

**Figure 3:**
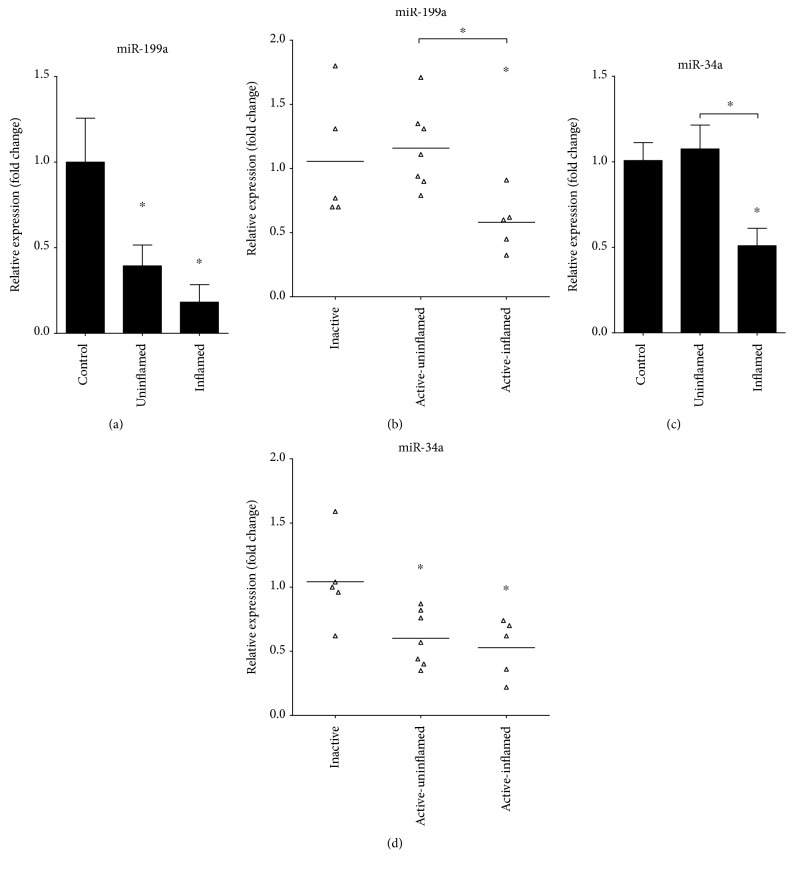
Decreased expression of miRNAs regulating Axl in inflamed colon. The relative expression of miR-199a (a, b) and miR-34a (c, d) is shown from rat (a, c) and human (b, d) samples. Data on panels (a) and (c) represent control (left columns, *n* = 2), uninflamed (middle columns, *n* = 6), and inflamed (right columns, *n* = 6) rat colon sections and are presented as the mean ± SEM; data on panels (b) and (d) represent inactive (left, *n* = 5), active-uninflamed (middle, *n* = 7), and active-inflamed (right, *n* = 5) colon samples of IBD patients where dots and lines represent individual values and the mean; ^∗^*p* < 0.05.

Altered expression of miRNAs is a hallmark of various cancer types. MicroRNAs regulating genes involved in EMT, such as miR-192 and miR-375, are downregulated in the inflamed colon regions in rat experimental colitis [32] and also in active-inflamed colons of IBD patients (Figure 2(l)). Importantly, microRNAs miR-199a and miR-34a, which posttranscriptionally regulate the expression of *AXL* [27–29], are also significantly downregulated in inflamed rat colons (Figures 3(a) and 3(c)) as well as in active-inflamed regions of IBD patients (Figures 3(b) and 3(d)). As a consequence, the posttranscriptional regulation of *AXL* is dysregulated; hence, its transcript (Figures 1(b) and 2(b)) and protein (Figure 1(i)) levels increase.

### 3.5. LPS Induces AXL Expression in THP-1 Monocytic Cell Line

**Figure 4:**
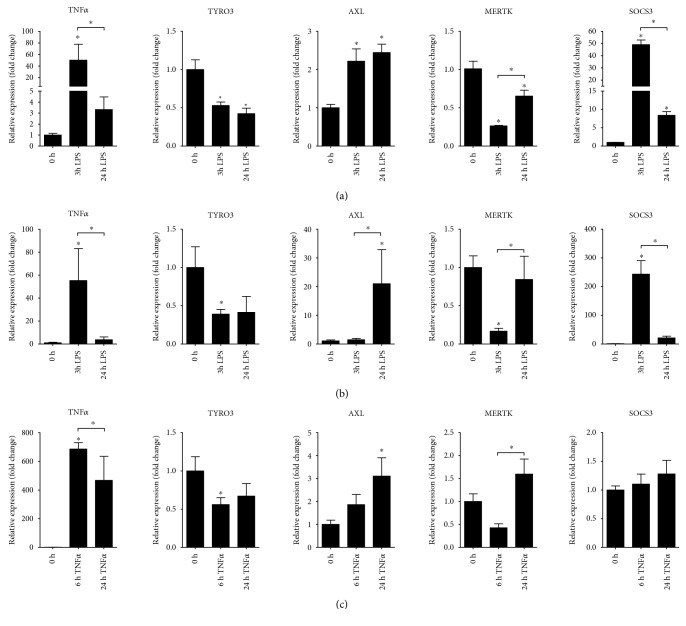
Distinct expression of TAM receptors in LPS triggered THP-1 and TNF*α* triggered HT-29 cells. The relative gene expression of the proinflammatory molecule *TNFα*, TAM receptors *TYRO3*, *AXL*, and *MERTK*, and the suppressor of cytokine signalling SOCS-3 in (a) inactivated LPS-stimulated THP-1, (b) PMA-activated LPS-stimulated THP-1, and (c) TNF*α*-stimulated HT-29 cells. Data are presented as the mean ± SEM; *n* = 3; ^∗^*p* < 0.05.

TAM receptors exhibit different tissue expression patterns, and their promoters are regulated by distinct extracellular stimuli [40, 41]. Next, we have used inactivated and PMA-activated THP-1 cell line of monocytic origin to determine if the LPS challenge alters the expression of TAM receptors. For this, we have stimulated the cells for 3 or 24 hours with LPS and have first determined the expression of *TNFα* as a positive control of induction. As expected, both inactivated and activated THP-1 cells responded with elevated expression of *TNFα* (Figures 4(a) and 4(b)). The expression of TAM receptors showed diverse expressions: while LPS downregulated the expression of *TYRO3* and *MERTK*, it significantly induced the expression of *AXL* (Figures 4(a) and 4(b)). Interestingly, while the expression of *AXL* in inactivated THP-1 cells shows modest induction at both 3 and 24 hours post LPS stimuli (Figure 4(a)), induction in activated THP-1 cells at 24 hours after LPS challenge is robust (Figure 4(b)). Since TAM receptors inhibit TLR-induced inflammation by the activation of, among others, SOCS3 anti-inflammatory molecule [13], we have next determined its expression. The expression pattern of *SOCS3* (Figures 4(a) and 4(b)) follows that of *TNFα* that is opposing expression pattern to that of *AXL* suggesting that even though increased *AXL* may act as a negative regulator of innate immunity upon LPS stimulation of THP-1 cells, the downstream effector molecule is probably not SOCS3.

### 3.6. TNF*α* Induces Gene Expression of AXL in HT-29 Colonic Epithelial Cell Line

**Figure 5:**
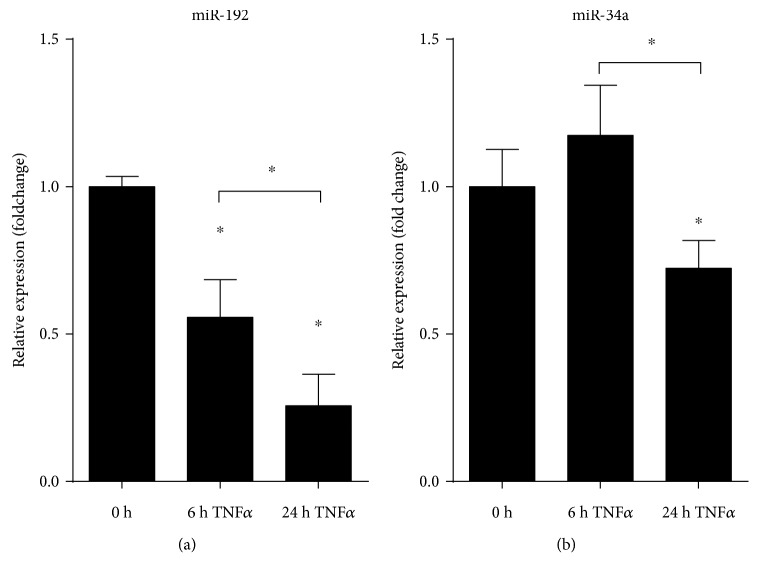
Decreased expression of miRNAs regulating Axl in TNF*α* triggered HT-29 colonic epithelial cells. The relative gene expression of miR-199a (a) and miR-34a (b) is shown from TNF*α* triggered HT-29 cells. Data are presented as the mean ± SEM; *n* = 3; ^∗^*p* < 0.05.

In order to determine if TAM receptors show altered expression in colonic epithelial cells, we have stimulated HT-29 colonic epithelial cells with TNF*α* for 6 or 24 hours and have again first monitored the expression of *TNFα* which is known to be self-induced. The robust expression of *TNFα* indicated successful induction of HT-29 cells (Figure 4(c)). *AXL* again showed induced expression at 24 hours postinduction (Figure 4(c)). Importantly, in contrast to THP-1 cells, the expression of *SOCS3* remained unaltered (Figure 4(c)) suggesting that the function of induced *AXL* in these cells is probably not the inhibition of inflammation, at least not through SOCS3. Finally, the expression of both miR-192 and miR-34a showed significant downregulation at 24 hours post-TNF*α* stimuli (Figures 5(a) and 5(b), resp.). These data are in complete agreement with those obtained from both rat and human colon samples indicating that TNF*α*-stimulated HT-29 cells are mimicking *in vivo* observations making them a perfect *in vitro* system for modelling the gene expression changes of colonic epithelial cells in IBD.

## 4. Conclusion

Our present study demonstrates that the epithelial-to-mesenchymal transition is triggered in IBD patients. These data are in agreement with our previous report showing the activation of EMT in rat experimental colitis [33]. In addition, we show that the increased expression of *AXL* is accompanied with the decreased expression of its microRNA regulators. These findings shed light onto a possible *AXL*- and microRNA-mediated regulation influencing epithelial-to-mesenchymal transition in IBD. In addition, by using *in vitro* monocytic and colonic epithelial cells, we show that upon stimulation with LPS or TNF*α*, respectively, the expression of *AXL* increases in both cell types.

Rothlin et al. in a recent review have underlined an apparently contradicting role of TAM receptors in the context of IBD and CRC, asking if individual members of the TAM family have tumor-promoting or antitumor effects [16]. The present study provides preliminary evidence on the possible oncogenic role of *AXL* in IBD: the increased expression of *AXL*—possibly due to the downregulated expression of its microRNA regulator/s, such as miR-199a and miR-34a—along with malfunctioning of the negative regulation of inflammation points toward *AXL* indeed being an important player in bridging IBD and CRC. Since the therapeutic targeting of AXL inhibits tumor growth and metastasis in ovarian cancer models [42], it is likely that AXL may become a potential therapeutic target in colitis-associated colorectal cancer.

## Figures and Tables

**Table 1:** SYBR Green primer sets used in QPCR experiments.

Gene	Forward (5′–3′)	Reverse (5′–3′)
CDH1	AGCCTGTCGAAGCAGGATTG	AGTCCTGGTCCTCTTCTCCG
MMP9	TCTATGGTCCTCGCCCTGAA	GCACAGTAGTGGCCGTAGAA
VIM	GGACCAGCTAACCAACGACA	AAGGTCAAGACGTGCCAGAG
HIF1*α*	GCCGCTGGAGACACAATCAT	CGTTTCAGCGGTGGGTAATG
ZEB2	CCAAGGAGCAGGTAATCGCA	ACGTTTCTTGCAGTTTGGGC
SNAI1	ACCCCAATCGGAAGCCTAAC	GGACAGAGTCCCAGATGAGC
Snai1	CGGAAGCCCAACTATAGCGA	AGAGTCCCAGATGAGGGTGG

**Table 2:** TaqMan Gene Expression Assays.

Gene	Assay number
Axl	Rn01457771_m1
Tyro3	Rn00567281_m1
Mertk	Rn00576094_m1
Pros1	Rn01527321_m1
Gas6	Rn00588984_m1
AXL	Hs00242357_m1
MERTK	Hs01031979_m1
TYRO3	Hs00170723_m1
PROS1	Hs00165590_m1
GAS6	Hs00181323_m1
18S RNA	Hs99999901_s1
TNF*α*	Hs00174128_m1
SOCS3	Hs01000485_g1

**Table 3:** miRNA-specific TaqMan microRNA assays.

miRNA	Assay number
RNU48	tm001006
U6	tm001973
miR-199a	tm002304
miR-192	tm000491
miR-34a	tm000426

## Data Availability

The authors have not used high-throughput analyses; hence, the data are not deposited.
